# Editorial: New Strategies for the Diagnosis and Treatment of Multi-Drug Resistant Bacteria or Fungi in Wounds

**DOI:** 10.3389/fmolb.2023.1248534

**Published:** 2023-07-28

**Authors:** Kok-Yong Chin

**Affiliations:** Department of Pharmacology, Faculty of Medicine, Universiti Kebangsaan Malaysia, Cheras, Malaysia

**Keywords:** chronic wound, wound healing, multi-drug resistant (MDR) bacteria, fungi, vaccines, antibiotics

Wounds that do not heal through the physiological process of normal wound healing are called chronic wounds (Ibrahim et al., 2018). Apart from the known modifiable and unmodifiable risk factors (Avishai et al., 2017), microorganisms appear to be a cause of delayed wound healing because they prolong the inflammation phase of wound healing. Bacteria and fungi, especially, form a biofilm constituted of multiple microorganisms which contribute to antimicrobial resistance (Zhao et al., 2013). Chronic wounds contribute to a significant healthcare burden. For example, surgical site infections are responsible for $3.3 billion in medical costs and 1 million additional inpatient days annually, according to the Centers for Disease Control and Prevention—National Healthcare Safety Network (Sen, 2021). Given the significance of this problem, this Research Topic aims to provide insight into the involvement of infection in chronic wounds, and the development of associated diagnostic and therapeutic innovations. We have received four submissions covering various on this Research Topic.

Wang et al. reviewed the classic and emerging technologies for identifying antimicrobial-resistant bacteria. The emergence of new technologies has shifted the identification procedures from broth dilution assay which is time-consuming and labour-intensive, to genomic, proteomic and metabolomic assays which are more precise and can determine the molecular mechanism of drug resistance. The advantages and limitations of each technology have been explained in detail for readers to make a fair comparison. The authors have recommended the combination of computational and multi-omics approaches in driving more accurate diagnosis, and machine learning for more precise surveillance of antimicrobial-resistant bacterial infection. In particular, the development of a database to identify markers of known antibiotic-resistance microorganisms and to advise the best treatment plan, as well as the use of artificial intelligence to predict unknown ones are emphasised.

Fungi are also a major contributor to delayed wound healing but the prevalence of this problem is grossly underestimated compared to bacterial etiology. Ge and Wang discussed the involvement of fungi, especially *Candida*, *Aspergillus, Trichophyton* and *Fusarium* species, in delayed wound healing. The role of fungi in shaping anti-microbial resistance at wound sites, for example, through the development of polymicrobial biofilm, is discussed. Due to polymicrobial involvement, quantitative molecular diagnostic methods for diagnosis and multimicrobial wound care protocols have been proposed. Treatment innovations, including nanomedicines, cell-based, biologics, hyperbaric oxygen, phototherapy and other microorganisms, in promoting chronic wound healing are also highlighted.

Vemula et al. employed computer modelling techniques to screen for a potential target against *Staphylococcus epidermidis*, a bacterial contributing to nosocomial infections through medical devices and surgical wounds (Wilson et al., 2005). They conducted molecular docking to screen 138 candidate molecules, calculated the binding energy of the molecules at the target sites using prime MM/GBSA analysis, predicted their absorption, distribution, metabolism, excretion and toxicity properties, and post MM/GBSA analysis in the process. They have identified several inhibitors to Ftz protein critical in bacterial cytokinesis are potential lead compounds for *S. epidermidis* infection.

This Research Topic also features an article by William et al. on developing a low-cost recombinant glycoconjugate vaccine against enterotoxigenic *Escherichia coli* (ETEC). Although ETEC is not responsible for chronic wound infection, other strains of *E. coli* are (Alharbi et al., 2019). In the study, they demonstrated site-specific installation of O-antigen polysaccharides (O-PS) corresponding to ETEC’s serogroups onto carrier proteins with oligosaccharyltransferase PglB from *Campylobacter jejuni*. Strong O-PS-specific humoral responses were elicited in mice by the conjugates. These reactions resulted in IgG antibodies with bactericidal activity against the associated infections. One of the prototype conjugates with serogroup O148 O-PS decreased ETEC colonization in mice, demonstrating mucosal protection. One would wonder if similar vaccines could be developed for *E. coli* strains related to wound infection.

As a conclusion, a multidisciplinary approach is required to tackle infection in delayed wound healing. Continuous innovations in the field of diagnostics, surveillance, and therapeutics are warranted, given the ever-changing landscape of multiresistant microorganisms.
